# New Appliances and Things Medical

**Published:** 1910-12-31

**Authors:** 


### NEW APPLIANCES & THINGS MEDICAL.

**[We shall be glad to receive at our Office, 28 & 29 Southampton Street, Strand, London, W.C., from the manufacturers, specimens of all new preparations and appliances.]**

### SKEFFINGTON'S PATENT LIFTER.

(TEMPORARY ADDRESS: 49 Ulundi Road, Blackheath, S.E.)

MR. ARTHUR SKEFFINGTON has kindly supplied us with a series of sketches showing the various methods in which this invalid lifter can be used. For institutions, as well as for private patients, the Skeffington Lifter is a most convenient apparatus; where the nurse is single-handed, the employment of such a labour-saving contrivance is indeed indispensable. To change the sheet without turning the patient on his side, to enable him to use the bed-pan comfortably, to slip under him air-rings or a water-bed without disturbing him in the manner that is now so commonly done when these necessary operations have to be performed—all this and many more simple and complicated performances the Skeffington may be relied upon to execute. The patent was awarded the silver medal at the Royal Sanitary Society's Brighton exhibition this year. Copies of the sketches and all particulars may be obtained on application to the makers.

